# The Pittsburgh Sleep Quality Index and Epworth Sleepiness Scale in frontotemporal dementia and Alzheimer's disease

**DOI:** 10.1002/alz.71686

**Published:** 2026-07-26

**Authors:** Jessica Jiang, Eva K Larsen, Benjamin A Levett, Lucy B Core, Sophie A Froud, Ciro della Monica, Hana Hassanin, Giuseppe Atzori, Chris JD Hardy, Marc A Busche, Derk‐Jan Dijk, Victoria L Revell, Samuel S Harris, Sofia H Eriksson, Jason D Warren

**Affiliations:** ^1^ Dementia Research Centre Queen Square Institute of Neurology University College London, London England UK; ^2^ Surrey Sleep Research Centre University of Surrey, Guildford England UK; ^3^ UK DRI Care Research and Technology Centre Imperial College London and University of Surrey, Guildford England UK; ^4^ Surrey Clinical Research Facility University of Surrey, Guildford England UK; ^5^ NIHR Royal Surrey CRF Royal Surrey NHS Foundation Trust, Guildford England UK; ^6^ UK Dementia Research Institute University College London London UK; ^7^ Department of Neurodegeneration University Hospital of Geriatric Medicine Felix Platter and Department of Biomedicine University of Basel Basel Basel‐Stadt Switzerland; ^8^ Department of Clinical and Experimental Epilepsy National Hospital for Neurology and Neurosurgery, London England UK

**Keywords:** alzheimer's disease, epworth sleepiness scale, frontotemporal dementia, pittsburgh sleep quality index, primary progressive aphasia, sleep

## Abstract

**INTRODUCTION:**

Sleep disturbance is common in dementia, impacting daytime function and care. Compared with Alzheimer's disease (AD), sleep in frontotemporal dementia (FTD) is poorly characterized.

**METHODS:**

We assessed sleep using the Epworth Sleepiness Scale and Pittsburgh Sleep Quality Index in 58 people with primary progressive aphasia (PPA) and right temporal variant FTD, 32 with AD, and 36 cognitively healthy older volunteers. All participants had cognitive and behavioral assessments. Groups were compared using non‐parametric statistics and correlations assessed sleep versus other indices.

**RESULTS:**

Subjective sleep duration was increased in all syndromic groups. AD and semantic PPA were associated with increased daytime somnolence. Reported sleep quality varied between syndromes. Across the disease cohort, somnolence correlated with behavioral and empathy deficits; in AD, poorer sleep quality correlated additionally with self‐monitoring deficits.

**DISCUSSION:**

FTD and AD syndromes have distinct sleep phenotypes, and sleep alterations are associated with behavior. Standard sleep scales require careful interpretation in dementia.

## Highlights

- We assessed sleep with standard scales in Alzheimer's and frontotemporal dementias.
- Sleep was prolonged in all syndromes.
- Increased daytime somnolence was frequent.
- Rated sleep quality varied across syndromes, perhaps based on homeostatic awareness.
- Sleep alterations correlated with general behavioral and empathy impairments.

## 1 BACKGROUND

Sleep is a core homeostatic function implicated as both a biomarker and driver of neurodegenerative proteinopathies, including Alzheimer's disease (AD) and frontotemporal dementia (FTD).^1^, ^2^, ^3^ These diseases target basal forebrain sleep circuitry early in disease progression and may establish a pathological “vicious cycle” by impairing restorative functions of sleep on synaptic and neural circuit integrity.^4^, ^5^, ^6^ Accordingly, there is considerable neurobiological and clinical interest in assessing sleep dysfunction in these diseases. However, their sleep phenotypes are poorly defined, particularly for FTD.^2^

FTD comprises a cluster of neurodegenerative diseases and syndromes, characterized by marked clinical, neuroanatomical, and histopathological heterogeneity^7^ but shared propensity to affect homeostatic functions^8^ and neural mechanisms relevant to sleep.^4^, ^8^, ^9^ There is accumulating evidence for clinically relevant sleep disturbances across the FTD spectrum.^2^, ^10^, ^11^ On clinical and neuroanatomical grounds, FTD syndromes are likely to have sleep phenotypes that are at least partly distinct from each other and from AD.^12^, ^13^ Sleep symptoms may occur earlier and more frequently in FTD than AD.^2^, ^12^ Moreover, because FTD syndromes are generally led by difficulties with social and emotional behavior and/or communication, sleep disturbances that impact these functions^14^, ^15^ are liable to have particularly severe functional consequences. However, data on sleep symptoms in FTD syndromes remain very limited, particularly in comparison to AD.

Here we addressed this in a cohort of patients representing major syndromes of FTD (the non‐fluent‐agrammatic and semantic variants of primary progressive aphasia [nfvPPA and svPPA], and right temporal lobe variant FTD [rtvFTD]) in relation to patients with typical AD and its language‐led variant (logopenic variant primary progressive sentence aphasia [lvPPA]) and cognitively healthy older individuals. We administered two standard, widely used sleep symptom scales: the Epworth Sleepiness Scale (ESS) and the Pittsburgh Sleep Quality Index (PSQI).^16^, ^17^ Our aims were to compare sleep symptom profiles in these FTD syndromes versus AD and to relate sleep indices to general cognitive and behavioral characteristics, including measures of daily‐life social functioning. Based on available evidence,^2^, ^11^, ^18^ we hypothesized that all dementia syndromes would be associated with increased sleep duration, with differentiable syndromic profiles of daytime somnolence and altered sleep quality.

## 2 METHODS

### 2.1 Participants

We assessed 32 participants with AD, 16 with lvPPA, 18 with nfvPPA, 15 with svPPA, nine with rtvFTD, and 36 cognitively healthy volunteers (Table 1). Patients with rtvFTD all presented with changes in social and emotional behavior fulfilling consensus diagnostic criteria for behavioral variant FTD^19^; all other patients met clinical criteria for their assigned syndromic diagnosis,^19^, ^20^, ^21^ of mild to moderate severity and with compatible brain magnetic resonance imaging (MRI) and AD biomarkers (all 29 of those tested with a clinical diagnoses of AD or lvPPA). One patient with AD (receiving treatment) and one with rtvFTD (untreated) had a prior diagnosis of obstructive sleep apnea; no other participant had a prior diagnosis of a clinical sleep disorder. All participants gave informed consent; ethical approval was granted by University College London and University of Surrey Research Ethics Committees, following Declaration of Helsinki guidelines.

**RESEARCH IN CONTEXT**

1. **Systematic review**: We reviewed relevant peer‐reviewed literature using online AD disease and frontotemporal dementia. The available evidence base is very limited.
2. **Interpretation**: Our findings, using standard sleep symptom scales (ESS and PSQI), show that people with FTD and AD have sleep alterations compared to cognitively healthy older people. Sleep profiles varied between dementia syndromes, encompassing increased overnight sleep duration, daytime somnolence, and altered sleep quality. Sleep alterations correlated with behavioral and empathy impairments.
3. **Future directions**: This study identifies a number of key areas for future exploration, including the use of parallel sleep symptom scales in patients and caregivers; correlation of sleep symptoms with objective measures of sleep physiology, such as actigraphy, polysomnography, oximetry, neurohormonal assays, functional neuroimaging, and assessments of daytime function; and engagement of larger, more diverse cohorts representing different disease stages.

**TABLE 1:** General demographic, clinical, behavioural and sleep characteristics of participant groups.

Characteristic	HV	AD	lvPPA	rtvFTD	svPPA	nfvPPA
**General**						
No.	36	32	16	9	15	18
Age	68.83 (6.69)	**73.94 (6.65)**	67.94 (7.97)^*^	69.67 (4.5)^*^	64.73 (7.89)^*^, ^^^	**72.17 (5.67)**
Sex (Male:Female)	16:20	15:17	12:4	8:1	7:8	10:8
Ethnicity (%): White/Other/Not specified	97/3/0	84/13/3	88/6/6	100/0/0	80/20/0	72/19/9
Symptom duration (years)	NA	5.09 (2.87)	4.88 (2.68)	6.22 (3.56)	4.27 (1.71)	3.82 (1.59)^a^
Medication use AD symptomatic meds^¶¶^	NA	30	15	NA	NA	NA
Sleeping meds^**^	0	0	1	0	1	0
MMSE (/30)	29 (1.19)	**18.81 (5.13)**	**16.77 (5.02)** ^c^	**25.11 (4.49)** ^*^, ^+^	**21.92 (8.15)** ^+^, ^b^	**23.17 (5.69)** ^*^, ^+^, ^f^
WASI Matrices score	26.25 (2.70)	**14.79 (8.58)**	**8.77 (5.59)** ^c^	23.38 (8.33)^*^, ^+^, ^^^, ^a^	21.79 (9.70)^*^, ^+^, ^^^, ^a^	**12.67 (7.23)** ^f^
**Behavior**						
CBI‐R score	NA	41.67 (27.04)	45.08 (21.16)^c^	48.67 (28.60)	45 (24.44)^b^	46.96 (42.03)^f^
**mIRI**: Total	NA	48.7 (10.81)^b^	44.86 (10.62)^b^	34.75 (8.96)^*^, ^+^, ^a^	40.62 (13.92)^*^, ^b^	43.43 (10.03)^d^
Cognitive empathy	NA	20.72 (6.31)	19.71 (5.65)^b^	15.13 (4.49)^a^	17.23 (7.56)^b^	19.07 (4.98)^d^
Emotional empathy	NA	27.43 (5.92)^b^	25.14 (5.91)^b^	19.63 (5.21)^*^, ^+^, ^a^	23.39 (7.18)^*^, ^b^	24.36 (6.03)^d^
**RSMS**: Total	NA	36.48 (15.02)^c^	35.85 (10.03)^c^	27.63 (11.30)^a^	29.77 (15.43)^b^	35.93 (13)^d^
Behavior sensitivity	NA	17 (7.55)^c^	16.69 (6.52)^c^	11.63 (7.39)^a^	12.69 (9.47)^b^	16.64 (6.72)^d^
Self‐monitoring	NA	19.55 (7.70)^a^	19.15 (4.53)^c^	16 (4.66)^a^	17.08 (6.18)^b^	19.29 (7.19)^d^
**Sleep**						
Average time (variation min): Retiring^¶^	22:45 (53)	22:42 (42)	22:09 (45)	22:18 (74)	22:14 (81)	22:29 (32)
Rising^¶^	07:16 (58)	**08:09 (73)**	07:50 (67)	08:10 (52)	07:36 (73)	07:18 (71)
Sleep latency (min)	20.46 (17.51)	16.73 (24.08)	8.36 (8.37)	11.33 (12.66)	36.29 (43.45)	26.38 (49.28)
Calculated sleep duration (h)	8.15 (1.09)	**9.14 (1.27)** ^i^	**9.66 (1.48)** ^e^	**9.75 (1.46)**	8.72 (1.43)^d^	8.43 (0.83)^h^
**ESS** Total	4.76 (4.32)	**8.62 (5.50)** ^b^	6.17 (3.65)^a^	6.89 (7.51)	**8.67 (7.05)**	7.33 (4.73)
Normal:Abnormal	32:4	20:10	13:2	7:2	11:4	15:3
**PSQI** Total	5.25 (2.87)	**3.64 (1.93)** ^g^	4.63 (2.90)	5.56 (2.60)	5.00 (4.29)	7.06 (4.82)^*^, ^b^
Normal:Abnormal	20:16	21:4	12:4	5:4	10:5	7:9
C1: Subjective sleep quality	7:26:3:0	**16:12:1:1**	5:8:3:0	3:5:0:1	**8:4:2:1**	4:9:2:2
C2: Sleep latency	15:14:3:4	13:14:1:1	10:5:1:0	7:1:0:1	8:4:1:2	7:4:2:4
C3: Sleep duration	15:12:8:1	19:8:1:0	10:3:30	7:2:0:0	11:2:1:1	8:6:2:1
C4: Sleep efficiency	18:11:5:2	13:12:2:1	13:1:1:1	2:2:3:2	11:2:0:2	6:6:3:2
C5: Sleep disturbances	0:28:8:0	0:24:5:0	0:8:8:0	0:6:3:0	0:12:3:0	0:8:7:2
C6: Sleep medication	33:3:0:0	30:1:0:0	14:1:0:1	9:0:0:0	12:1:0:2	12:1:2:2
C7: Daytime dysfunction	17:16:3:0	12:18:1:0	7:8:1:0	1:6:2:0	6:7:1:1	3:11:2:0

*Note*: Mean and standard deviations (SD) are reported unless otherwise indicated. Numbers of individuals in each group falling into each PSQI component (C) category are presented, coded as follows: **C1**, Very good: Fairly good: Fairly bad: Very bad; **C2**, Less than 15 min: 16 to 30 min: 31 to 60 min: Over 60 mins; **C3**, Over 7 h: 6 to 7 h: 5 to 6 h: Less than 5 h; **C4**, Over 85% efficiency: 75% to 84% efficiency: 65% to 74% efficiency: Less than 65% efficiency; **C5**, Very little: Fairly little: Fairly more: Many; **C6**, Question was: “During the past month, how often have you taken medicine (prescribed or ‘over the counter’) to help you sleep?” Response options: Not during the past month: Less than once a week: Once or twice a week: Three or more times a week; **C7**, No problem at all: Only a very slight problem: Somewhat of a problem: A very big problem. Significant values are coded as follows: **bold**, significantly different from cognitively healthy volunteers.

^¶^ Based on 24‐h clock.

* Significantly different from AD.

^+^ Significantly different from lvPPA.

^^^ Significantly different from nfvPPA.

^¶¶^ Donepezil and/or memantine.

** Melatonin, zolpidem, diphenhydramine. Numbers of missing participant datasets coded as follows:

^a^ 1 missing,

^b^ 2 missing,

^c^ 3 missing,

^d^ 4 missing,

^e^ 5 missing,

^f^ 6 missing,

^g^ 7 missing,

^h^ 8 missing,

^i^ 12 missing.

Abbreviations: AD, patient group with typical Alzheimer's disease; CBI‐R, Cambridge Behavioural Inventory (Revised); ESS, Epworth Sleepiness Scale; lvPPA, patient group with logopenic variant primary progressive aphasia; mIRI, modified Interpersonal Reactivity Index; MMSE, Mini‐Mental State Examination; NA, not applicable; nfvPPA, patient group with non‐fluent variant primary progressive aphasia; PSQI, Pittsburgh Sleep Quality Index; RSMS, Revised Self‐Monitoring Scale; rtvFTD, patient group with right temporal variant frontotemporal dementia; svPPA, patient group with semantic variant primary progressive aphasia; WASI, Wechsler Abbreviated Scale of Intelligence.

### 2.2 Sleep assessments

All participants estimated their usual times to retire and to rise from bed and how long it took them to fall asleep (i.e., sleep latency); average night‐time sleep “duration” was calculated for each participant by subtracting estimated latency to sleep onset. Information for patients was provided by their caregivers; in each case, the caregiver had close knowledge of the patient's sleep and circadian behavior patterns and was their long‐term bed partner in most cases. Cognitively healthy volunteers reported on their own sleep.

To score sleep symptoms, we used the ESS and PSQI.^16^, ^17^ Patients’ primary caregivers completed the questionnaires on their behalf; cognitively healthy volunteers completed their own questionnaires. All sleep questionnaires and other cognitive and behavioral assessments were completed on the same day.

The ESS is used to quantify unusual daytime sleepiness, often in association with narcolepsy, obstructive sleep apnea or other disruptive sleep phenotypes.^17^ It presents eight everyday scenarios and scores each (on a scale from 0 to 3) for how likely the assessed person was likely to doze off or fall asleep during the past week in that scenario, a score of 0 indicating they “would never doze off” and 3 a “high chance of dozing” (maximum total score 24). A score >10 suggests unusual levels of daytime sleepiness.

The PSQI is used to assess night‐time sleep quality over the past month in order to identify the presence of sleep disorders.^16^ It comprises 19 items covering seven subcategories: sleep duration, subjective sleep quality, sleep latency, use of sleeping medication, daytime dysfunction, sleep disturbances, and habitual sleep efficiency. Scores across items sum to a maximum total score of 21; a score >5 suggests poor sleep quality.

In addition to the Mini‐Mental State Examination (MMSE), an index of overall cognitive function, and the Wechsler Abbreviated Scale of Intelligence (WASI) Matrices test, an index of executive function, we administered validated scales of everyday socio‐emotional behavior and awareness in dementia: the Cambridge Behavioural Inventory (CBI) (Revised),^22^ the modified Interpersonal Reactivity Index (mIRI), and Revised Self‐Monitoring Scale (RSMS) questionnaires.^23^, ^24^

### 2.3 Analyses

Data were analyzed in R (version 4.1.2). For continuous demographic, cognitive, and questionnaire data, participant groups were compared using one‐way ANOVA or Kruskal‐Wallis tests, based on the fulfillment of normality (Shapiro Wilk). For clock‐time data, the Watson‐Williams test was used. For PSQI and ESS scores, an ordinal logistic regression (proportional odds) was conducted to examine associations between diagnosis and sleep measures; the outcome variable (PSQI or ESS total score) was treated as ordinal, and the predictor of interest was diagnosis, with age included as a covariate. To compare PSQI and ESS profiles, raw scores were first converted to z‐scores based on mean scores for the cognitively healthy group. Group categorical data were compared initially using an omnibus Fisher's exact test, to assess overall group differences in response rates. Pairwise Fisher's exact tests were subsequently conducted between diagnostic groups, to explore specific contrasts. *P* values from pairwise tests (including post hoc tests) were adjusted for multiple comparisons using the Benjamini‐Hochberg method. Spearman's rank correlation was used to assess how sleep symptom scores related to age, symptom duration, and MMSE, WASI Matrices, CBI, mIRI, and RSMS scores, over the whole patient cohort and in the largest, AD patient group. A statistical significance threshold of *p* < 0.05 was accepted for all tests.

## 3 RESULTS

### 3.1 General participant group characteristics

Participant groups did not differ significantly in sex distribution but did differ in age (χ^2^[5] 20.282, *p* = 0.001): The AD and nfvPPA groups were significantly older than the svPPA and cognitively healthy volunteer groups, and the AD group was additionally significantly older than the lvPPA and rtvFTD groups (Table 1). Patient groups did not differ significantly in symptom duration (*p* = 0.306) but did differ in general cognitive function: on MMSE (χ^2^[4] 16.78, *p* = 0.002), the AD and lvPPA groups performed significantly worse than the rtvFTD, svPPA, and nfvPPA groups. Patient groups did not differ significantly for CBI or RSMS scores but did differ in mIRI total score (*F*[4,74] 3.07, *p* = 0.021) and emotional empathy subscale (χ^2^[4] 10.60, *p* = 0.031): The rtvFTD and svPPA group scored significantly lower than the AD group, and the rtvFTD group additionally scored significantly lower than the lvPPA group (Table 1).

### 3.2 Sleep assessments

Participant groups differed significantly in calculated estimated hours usually slept overnight (*F*[5,91] 4.61, *p* = 0.001) (Table 1). Relative to the cognitively healthy group, significantly longer mean calculated overnight sleep durations were recorded for the groups with AD (*t* = 2.87, *p* = 0.026), lvPPA (*t* = 3.55, *p* = 0.006), and rtvFTD (*t* = 3.48, *p* = 0.006); average time of rising was significantly later in the AD group (*F* = 10.44, *p* = 0.030).

**FIGURE 1:**
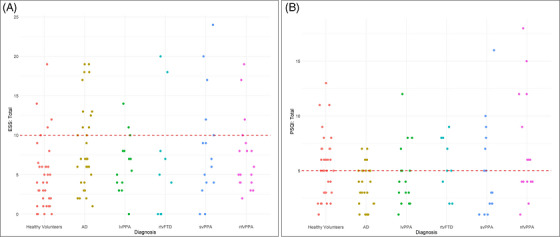
Scatter plot of Epworth Sleepiness Scale (ESS) and Pittsburgh Sleep Quality Index (PSQI) scores across participant groups. (A) ESS: red line (>10) indicates abnormal sleepiness score. (B) PSQI: red line (>5) indicates abnormal sleep quality score. AD, patient group with typical Alzheimer's disease; lvPPA, patient group with logopenic variant primary progressive aphasia; nfvPPA, patient group with nonfluent variant primary progressive aphasia; rtvFTD, patient group with right temporal variant frontotemporal dementia; svPPA, patient group with semantic variant primary progressive aphasia.

**FIGURE 2:**
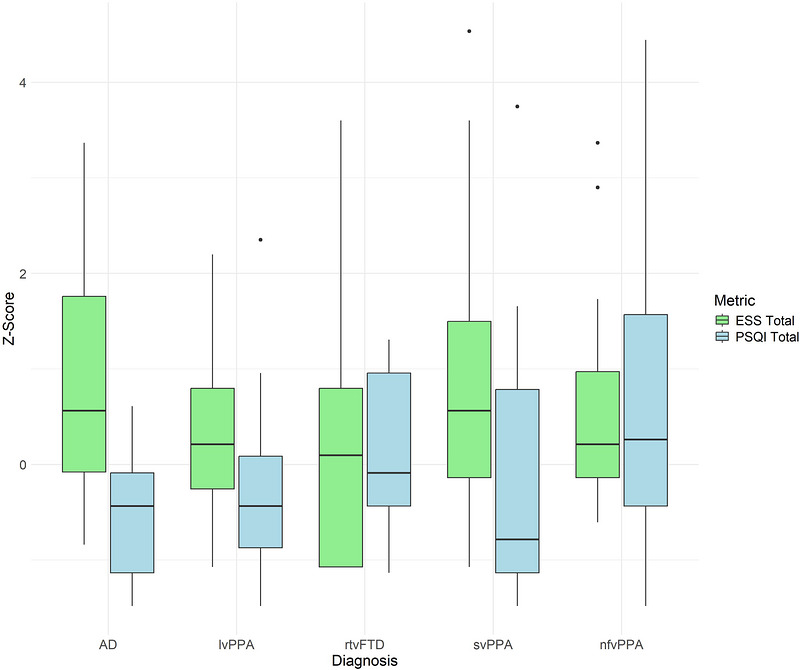
Box plots of ESS and PSQI z‐scores across patient participant groups. Blue boxes represent PSQI total z‐scores, green boxes the ESS total z‐scores. AD, patient group with typical Alzheimer's disease; ESS, Epworth Sleepiness Scale; lvPPA, patient group with logopenic variant primary progressive aphasia; nfvPPA, patient group with non‐fluent variant primary progressive aphasia; PSQI, Pittsburgh Sleep Quality Index; rtvFTD, patient group with right temporal variant frontotemporal dementia; svPPA, patient group with semantic variant primary progressive aphasia.

Participant groups were borderline significant overall on ESS score (χ^2^[5] 10.92, *p* = 0.053) (Table 1, Figure 1): Patients with AD (odds ratio [OR] 3.50, 95% confidence interval [1.42, 2.16], *p* = 0.006) and svPPA (OR 4.18 [1.36, 12.83], *p* = 0.013) had significantly higher scores (i.e., more daytime somnolence) than cognitively healthy volunteers. Participant groups did not differ significantly overall in PSQI total score (χ^2^[5] 10.00, *p* = 0.075 (Table 1, Figure 1). The AD group had significantly lower scores (i.e., better night‐time sleep quality) than the cognitively healthy group (OR 0.32 [0.13, 0.83], *p* = 0.0191) and nfvPPA group (OR 0.21 [0.07, 0.67], *p* = 0.008). Examining PSQI component scores (Table 1), there was a significant overall group difference for subjective sleep quality (*p* = 0.030), habitual sleep efficiency (*p* = 0.038), and sleep disturbances (*p* = 0.049). For sleep quality, the AD group was significantly different (*p* = 0.049) and the svPPA group borderline significantly different (*p* = 0.059) from the cognitively healthy group. The difference between ESS and PSQI z‐scores was significant for the AD group (*V* = 8, *p* < 0.001) and borderline for the lvPPA group (*V* = 26, *p* = 0.057) (Figure 2).

Over the combined patient cohort, ESS total score was significantly correlated with CBI‐R (*R* 0.38, *p* < 0.001) and mIRI empathy subscale (*R* −0.24, *p* = 0.036) scores (Figure S1). In the AD group, ESS total score was significantly correlated with CBI‐*R* (*R* 0.48, *p* = 0.007), mIRI total (*R* −0.39, *p* = 0.038), and empathy subscale (*R* ‐0.53, *p* = 0.004) scores; PSQI total score was significantly correlated with CBI‐R (*R* 0.47, *p* = 0.018), mIRI total (*R* −0.45, *p* = 0.030), mIRI empathy subscale (*R* −0.56, *p* = 0.005), RSMS total (*R* −0.45, *p* = 0.027), monitoring subscale (*R* −0.44, *p* = 0.031), and sensitivity subscale (*R* −0.47, *p* = 0.020) scores (Figure S2).

## 4 DISCUSSION

Our findings show that people with FTD and AD syndromes exhibit altered and heterogeneous sleep phenotypes relative to cognitively healthy older people. Compared to cognitively healthy volunteers, mean calculated overnight sleep duration was longer in all syndromic groups. Sleep symptom profiles differed between syndromes, as assessed on widely used sleep symptom scales. People with AD had more daytime somnolence but better sleep quality than cognitively healthy older people and patients with nfvPPA. svPPA was associated with increased daytime somnolence. Daytime somnolence was correlated with general behavioral and empathy impairments across the patient cohort; in people with AD, poorer sleep quality was associated with reduced empathy and self‐monitoring. These findings corroborate previous evidence for increased daytime sleepiness in dementia^25^ and distinct profiles of sleep symptoms across FTD and AD.^2^, ^11^, ^18^ This study extends previous work^26^, ^27^, ^28^, ^29^ in comparing FTD and AD directly on two widely used clinical scales of sleepiness and sleep quality, the ESS and PSQI.

Increased time asleep and excessive daytime sleepiness have been described in AD.^2^, ^18^, ^26^, ^30^, ^31^, ^32^ These symptoms impact daytime functioning,^33^ though in general they are less salient than in dementia with Lewy bodies. The finding of better overall sleep quality (lower PSQI scores) in the AD group versus cognitively healthy volunteers initially seems somewhat paradoxical; while this could in part reflect longer sleep durations (since the PSQI scoring does not account for pathologically “excessive” overnight sleep), subjective sleep quality was also rated as “very good” in a higher proportion of the AD group.^34^ This lower PSQI score contrasted sharply with the higher daytime sleepiness (ESS) score in the AD group (Figure 2): While this could imply that the ESS is easier to “objectify” for caregivers, a similar profile was observed previously in AD using patients’ own ratings, modulated by illness duration and/or severity as well as cultural factors.^35^, ^36^, ^37^, ^38^ Moreover, the AD profile is echoed here in the lvPPA (AD language variant) group but not in other syndromic groups (Table 1, Figure 2) and was previously reported in incipient AD dementia,^39^ suggesting that it may be a neurobiological signal of AD pathology. This finding speaks to a potential mismatch between subjective and objective indices of sleep disturbance, a wider issue in AD as well as FTD and other dementia syndromes,^27^, ^36^, ^37^, ^39^, ^40^, ^41^ which may reflect impaired awareness of homeostatic states in neurodegenerative proteinopathies that target interoceptive mechanisms.^4^, ^6^, ^9^, ^18^

Sleep disturbance is a significant issue in FTD syndromes, but it is much less well studied than in AD.^2^, ^11^, ^42^ Our findings of increased overnight sleep and increased daytime sleepiness in rtvFTD and PPA syndromes are broadly in line with available evidence.^2^, ^11^ Sleep phenotypes remain poorly defined in the FTD spectrum. While we did not replicate the greater prominence of sleep symptoms in rtvFTD compared with svPPA reported in some previous work,^43^ this may depend on the relative extent of bilateral temporal lobe involvement. Our finding of reduced sleep quality in nfvPPA fits with emerging evidence both in humans and mouse models linking 4‐repeat tauopathy (the predominant molecular substrate in nfvPPA) to sleep disturbances.^44^, ^45^

Our findings further underline that sleep disturbance impacts daily‐life socio‐emotional functioning and behavior in dementia syndromes. In the AD group, there were additional correlations with self‐monitoring capacity. Sleep disturbance may become prominent earlier and may therefore be less likely to track overall disease severity in FTD syndromes than in AD.^43^, ^46^ It is likely that sleep quality impacts the effectiveness of cognitive and behavioral interventions for dementia.^42^, ^47^

This study has several limitations that should guide further work. The findings should be corroborated with objective sleep measures, such as actigraphy and particularly electroencephalogram polysomnography and oximetry, to define sleep‐phase physiology and sleep‐related breathing disorders. While we did not find evidence for sleep disturbances or medication use driving sleep symptoms here, these factors warrant more detailed exploration.^18^ Collection of sleep scales such as ESS and PSQI and other measures of daily‐life functioning over the whole circadian cycle in parallel from patients and caregivers, ideally with physiological measures of interoception, would further help to determine the extent to which such measures are affected by subjective awareness of sleep symptoms. The sources of the substantial individual variability within diagnostic groups here should be clarified,^32^ including the role of disease severity. While there is no single satisfactory severity measure for all these dementia syndromes, measures of everyday functioning (such as Clinical Dementia Rating), and caregiver burden would be pertinent.^48^ Given the relatively small cohort size here, our findings should be extended in future collaborative, cross‐center studies to larger and more diverse cohorts and stratified by disease stage, which would likely modify sleep phenotypes considerably.^30^ Ultimately, a mechanistic understanding of sleep alterations in neurodegenerative syndromes will depend on correlating clinical phenotypes with physiological, molecular, and neurohumoral markers and functional neuroanatomy.^4^, ^6^, ^9^, ^11^, ^18^, ^49^

This work has highlighted potential shortcomings of standard sleep scales when it comes to fully characterizing sleep symptoms in people with dementia. The PSQI, for example, does not code pathologically increased overnight sleep times, reduced sleep latencies, or sleep attacks potentially relevant in FTD.^50^ The ESS might be more useful as a validated sleep scale in these syndromes. Clearly, a detailed sleep history should be obtained for all people with AD and FTD.

Taken together, our findings suggest that sleep scales such as ESS and PSQI may be useful in stratifying sleep profiles in different dementia syndromes and for predicting daily‐life behaviors and emotional awareness. Wider use of such scales could be useful in both clinical practice and, potentially, trials involving people with FTD and AD. However, care is needed in interpreting sleep scales clinically in dementia. Future work should address the factors that drive sleep symptom profiles in these diseases.

## CONFLICT OF INTEREST STATEMENT

The authors declare no conflicts of interest. Author disclosures are available in the Supporting Information.

## CONSENT STATEMENT

All human subjects provided informed consent

## Supporting information




Supporting Information

Supporting Information
