# Supplementary material for: The Pittsburgh Sleep Quality Index and Epworth Sleepiness Scale in frontotemporal dementia and Alzheimer's disease
Source: Alzheimers Dement. 2026 Jul 26;22(7):e71686. doi: 10.1002/alz.71686 (PMC13402253; doi:10.1002/alz.71686)
Supplement: Supplementary file 1 — Supporting Information [file ALZ-22-e71686-s001.docx]

**Supplementary Materials: Jiang et al., Sleep symptom profiles in frontotemporal dementia and Alzheimer’s disease**

**by J Jiang et al**

**
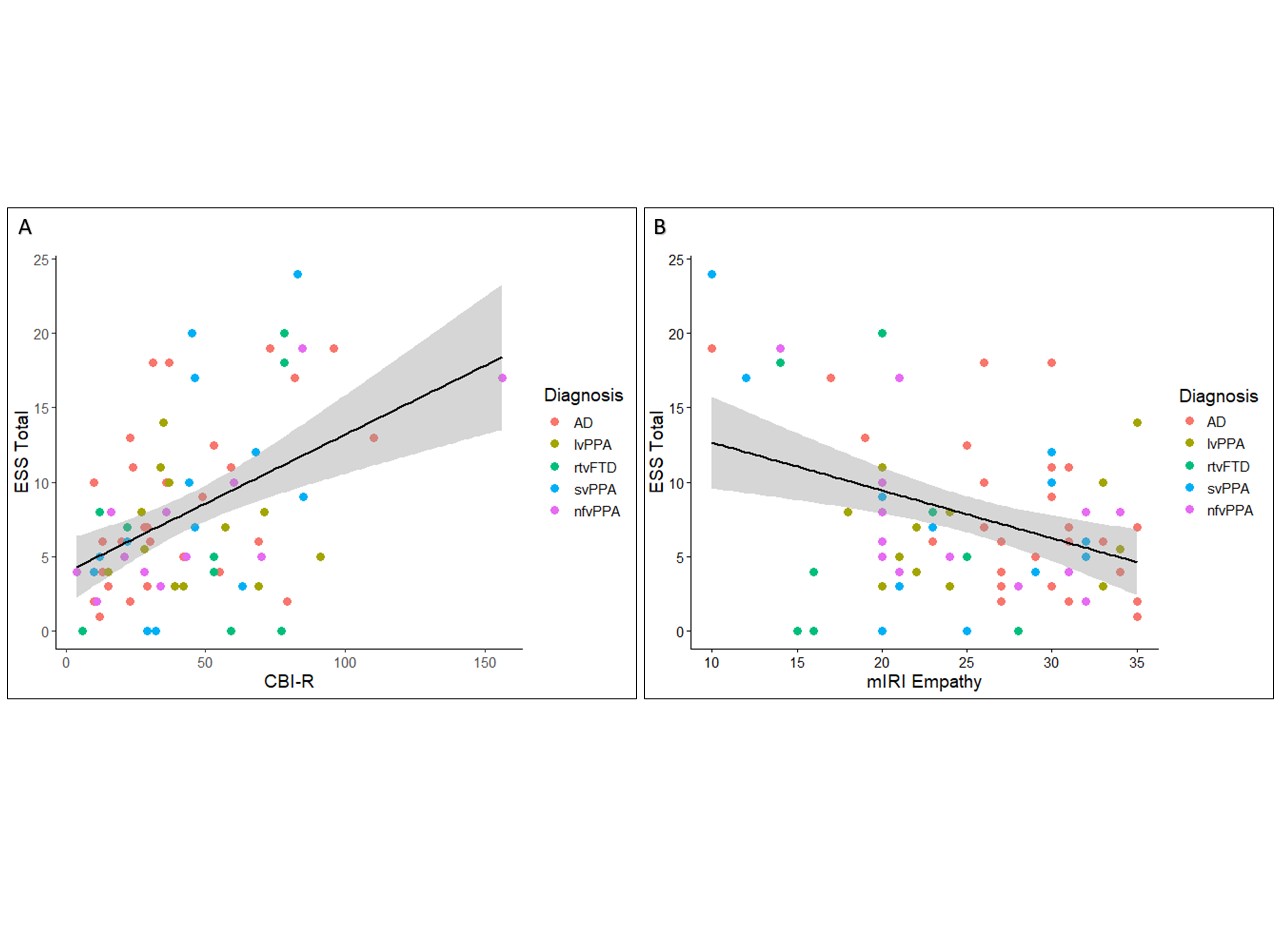
**

**Figure S1. Correlation plots of Epworth Sleepiness Scale and behavioural measures across all patient participant groups.** A) Correlation plot between Epworth Sleepiness Scale (ESS) and Cambridge Behavioural Inventory Revised (CBI-R); B) Correlation plot between ESS and the modified Interpersonal Reactivity Index (mIRI) empathy subscale. Red dots represent participants with Alzheimer’s disease (AD); yellow dots represent participants with logopenic variant primary progressive aphasia (lvPPA); green dots represent participants with right temporal variant frontotemporal dementia; blue dots represent semantic variant primary progressive aphasia; purple dots represent nonfluent variant primary progressive aphasia.

**
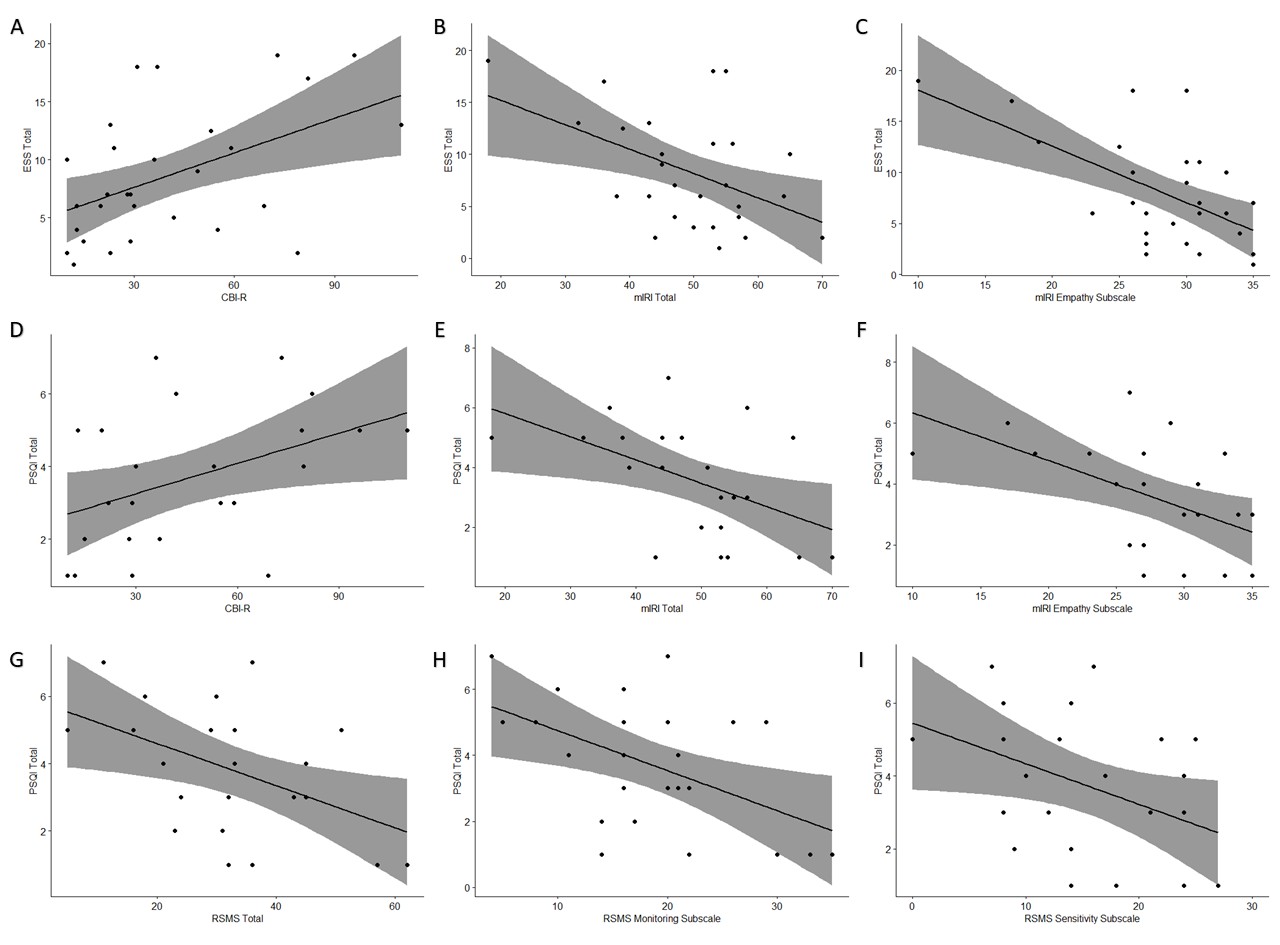
**

**Figure S2. Correlation plots of Epworth Sleepiness Scale and Pittsburgh Sleep Quality Index against cognitive and behavioural measures across the AD participant group.** A) Correlation plot between Epworth Sleepiness Scale (ESS) and Cambridge Behavioural Inventory Revised (CBI-R); B) Correlation plot between ESS and the modified Interpersonal Reactivity Index (mIRI); C) Correlation plot between ESS and the modified Interpersonal Reactivity Index (mIRI) empathy subscale; D) Correlation plot between Pittsburgh Sleep Quality Index (PSQI) and the CBI-R; E) Correlation plot between PSQI and the mIRI; F) Correlation plot between PSQI and the mIRI empathy subscale; G) Correlation plot between PSQI and Revised Self-Monitoring Scale (RSMS); H) Correlation plot between PSQI and RSMS monitoring subscale; I) Correlation plot between PSQI and RSMS sensitivity subscale.
